# Supplementary material for: Adaptation to sub-optimal hosts is a driver of viral diversification in the ocean
Source: Nat Commun. 2018 Nov 8;9:4698. doi: 10.1038/s41467-018-07164-3 (PMC6224464; doi:10.1038/s41467-018-07164-3)
Supplement: Supplementary file 1 — Supplementary Information [file 41467_2018_7164_MOESM1_ESM.pdf]

Supporting Figures and Tables to Enav et al.

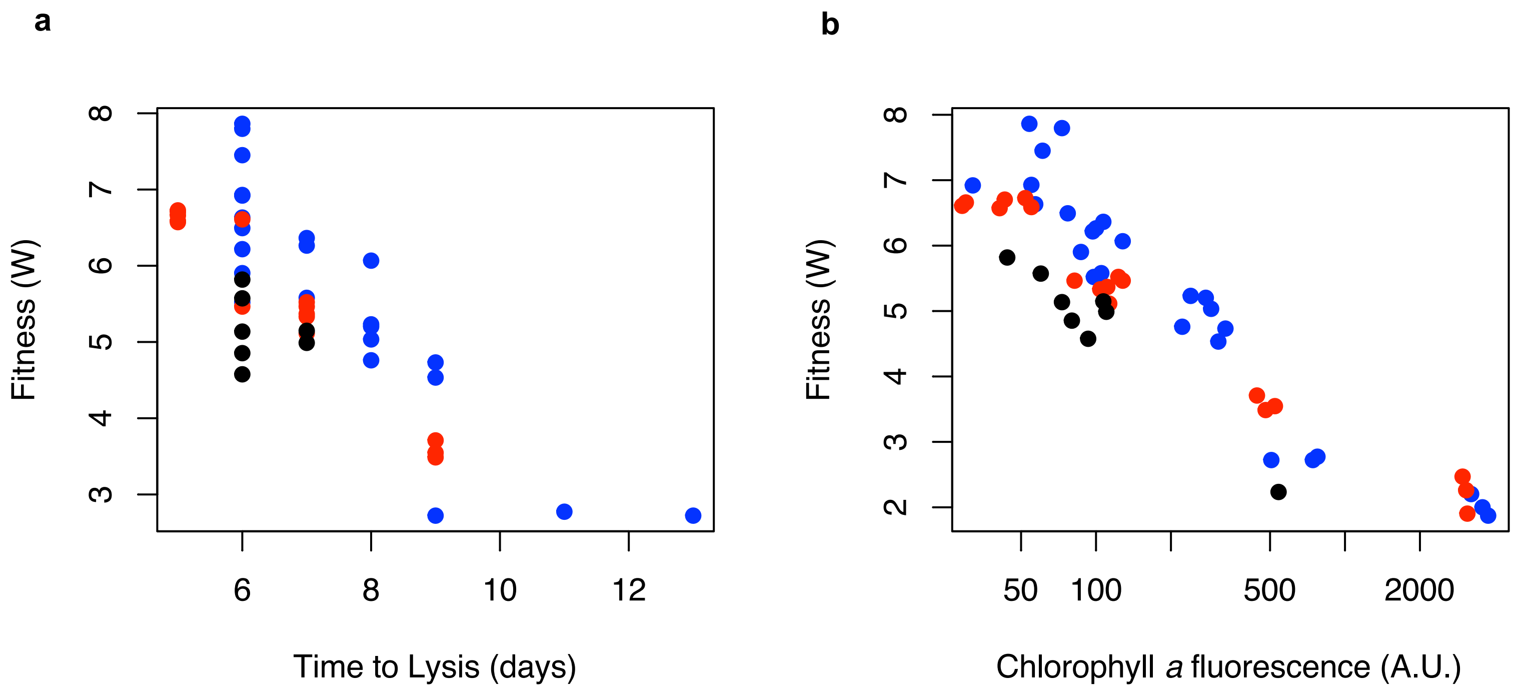

Supplementary Figure 1. Correlation between viral fitness and host lysis in a cyanophage-cyanobacterial system. Host lysis and fitness of wild-type and mutant P-SSP7 cyanophages infecting two strains of *Prochlorococcus* MED4 are shown using data from Schwartz et al.[1] (see Figure 2a and Supplementary Figure S1 of that manuscript). Three independent replicate experiments are presented with n=26 (experiment 1, blue circles), n=18 (experiment 2, red circles) and n=8 (experiment 3, black circles). Fitness was defined as the production of phage progeny over multiple rounds of infection and is presented as W, the number of population doublings per day. **a.** Fitness of each virus was plotted against the time to complete lysis of *Prochlorococcus* MED4 in each interaction. An infected culture was considered to be

completely lysed when chlorophyll *a* fluorescence readings were close to background levels (<100 A.U.). Six infected cultures, for which complete lysis was not reached during the time frame of the experiment, were excluded from these analyses. The Spearman's *rho* values are -0.87, -0.91, -0.38 and -0.84 for experiment 1, 2, 3 and for all 3 experiments combined, respectively. **b**. Fitness of the different viruses was plotted against chlorophyll *a* fluorescence of infected *Prochlorococcus* MED4 cultures 6 days after infection. A.U. – arbitrary units. Spearman correlation analysis was conducted for each independent experiment and for all experiments combined (*rho* = -0.95, -0.91, -0.74 and -0.89 for experiment 1, 2, 3 and for all 3 experiments combined, respectively). These findings, together with those of Turner et al.[26], indicate that a negative correlation between viral fitness and time to lysis is likely to be a general phenomenon that spans vastly different host-virus systems. They further show that host density at a set time after infection is a suitable proxy for phage fitness, and was used in this study.

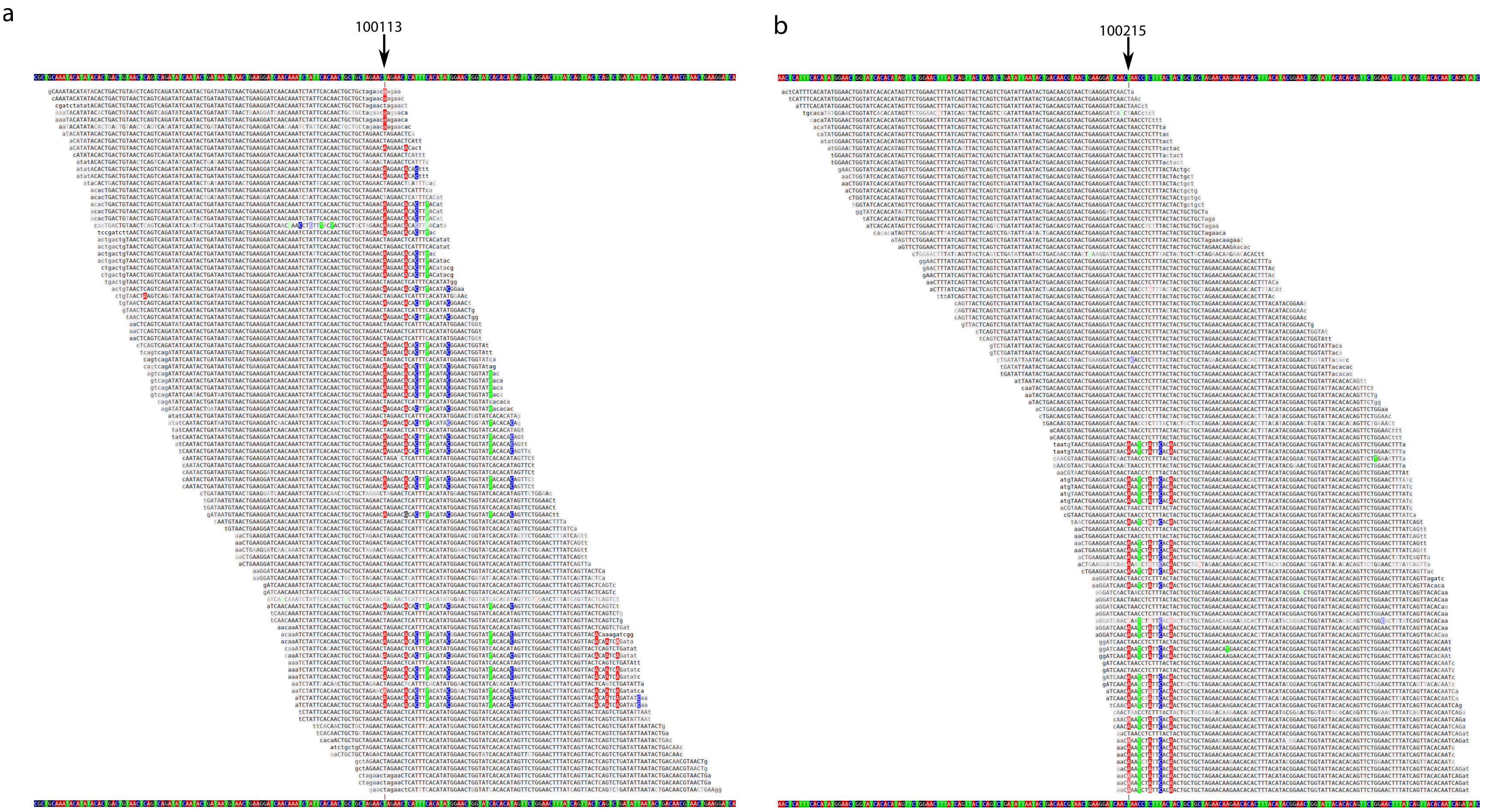

Supplementary Figure 2. Codon optimizing mutations are clustered together in specific phage genomes. View of representative raw DNA reads, from population #3, evolved in WH8102 host. Highlighted bases represent mutations. Arrows on top show the genomic position of the central base in each genomic region view. (**a**) Region flanking genomic

position 100113. The sequence of the mutated region is identical to that of the region between genomic positions 100242-100308, (**b**) Region surrounding position 100215. Mutated sequence is identical to the region between positions 10078-10098.

Supplementary Table 1: Normalized cell density of cyanobacterial cultures, infected by the ancestral S-TIM4 phage, 10 days post infection

**Host**	**Average normalized cell density (infected/control)**	**Std. deviation**
MIT9515	$5.26 * 10^{-3}$	$1.6 * 10^{-3}$
MED4	$7.35 * 10^{-2}$	$3.0 * 10^{-3}$
WH8102	$4.0 * 10^{-1}$	$8.7 * 10^{-2}$

* Obtained using *chlorophyll a* fluorescence readings.

Supplementary Table 2 : Summary of mutations identified in S-TIM4 populations

Genomic position	Population	Type	frequency	old base (SNP)	new base (SNP)	old AA (SNP)	New AA (SNP)	Synonymous (yes/no)	Codon adapting	Deleted/Inserted sequence	InDel length	metagenomic Island	mutation location in the gene	ORF
5285	MED4_1	SNP	0.0337	A	C	S	G	N	-		NA	y	661	13
16545	WH8102_4	SNP	0.1144	C	T	P	L	N	-		NA	n	65	23
16548	WH8102_4	DEL	0.4489	NA	-	NA	-	-	-	ATGAATTATGTGAT AGAACCATA	23	n	68	23
16941	WH8102_4	DEL	0.1969	NA	-	NA	-	-	-	AGAAAGGGGACAT CGTAATCTTCCCT	26	n	461	23
16945	WH8102_3	INS	1	NA	-	NA	-	-	-	G	1	n	465	23
17215	WH8102_1	SNP	0.3607	T	C	S	P	N	-		NA	n	22	24
17230	WH8102_1	SNP	0.1017	C	T	P	S	N	-		NA	n	37	24

17309	WH8102_1	SNP	0.4408	C	T	A	V	N	-		NA	n	116	24
17378	WH8102_4	SNP	0.0623	T	C	L	S	N	-		NA	n	185	24
17441	WH8102_3	SNP	0.0468	A	G	Y	C	N	-		NA	n	248	24
17452	WH8102_4	INS	0.1618	NA	-	NA	-	-	-	GTAACTCATATGGT GAGTTTG	21	y	259	24
17694	WH8102_1	SNP	0.0259	G	A	W	*	N	-		NA	y	501	24
17806	MED4_1	SNP	1	G	A	G	R	N	-		NA	y	613	24
17806	MED4_2	SNP	1	G	A	G	R	N	-		NA	y	613	24
17806	MED4_3	SNP	1	G	A	G	R	N	-		NA	y	613	24
17806	MED4_4	SNP	1	G	A	G	R	N	-		NA	y	613	24
17806	MED4_5	SNP	1	G	A	G	R	N	-		NA	y	613	24
17806	MIT9515_1	SNP	0.6492	G	A	G	R	N	-		NA	y	613	24
17806	MIT9515_2	SNP	0.7949	G	A	G	R	N	-		NA	y	613	24
17806	MIT9515_3	SNP	0.6598	G	A	G	R	N	-		NA	y	613	24
17806	MIT9515_4	SNP	0.8008	G	A	G	R	N	-		NA	y	613	24
17806	WH8102_1	SNP	1	G	A	G	R	N	-		NA	y	613	24
17806	WH8102_2	SNP	1	G	A	G	R	N	-		NA	y	613	24
17806	WH8102_3	SNP	0.9595	G	A	G	R	N	-		NA	y	613	24
17806	WH8102_4	SNP	1	G	A	G	R	N	-		NA	y	613	24
17831	WH8102_4	SNP	0.0450	G	A	W	*	N	-		NA	y	638	24
18024	WH8102_2	SNP	1	G	A	W	*	N	-		NA	y	114	25
18203	WH8102_4	SNP	0.0256	G	A	G	D	N	-		NA	n	293	25
19204	WH8102_3	SNP	0.0730	A	G	K	R	N	-		NA	n	473	27
19342	WH8102_3	SNP	0.5465	T	C	V	A	N	-		NA	n	611	27
19939	WH8102_1	SNP	0.0379	A	G	Q	R	N	-		NA	n	1208	27
20534	WH8102_3	SNP	0.0190	C	G	S	R	N	-		NA	y	1803	27
24475	MED4_1	SNP	0.0726	C	T	A	V	N	-		NA	n	764	34
24475	WH8102_1	SNP	0.1754	C	T	A	V	N	-		NA	n	764	34
24478	WH8102_4	SNP	0.3307	A	T	E	V	N	-		NA	n	767	34
25423	MIT9515_1	SNP	0.2290	G	A	G	D	N	-		NA	n	404	35
25423	MIT9515_2	SNP	0.09999	G	A	G	D	N	-		NA	n	404	35
25423	MIT9515_3	SNP	0.2094	G	A	G	D	N	-		NA	n	404	35
25423	MIT9515_4	SNP	0.0881	G	A	G	D	N	-		NA	n	404	35
25423	WH8102_1	SNP	0.1711	G	A	G	D	N	-		NA	n	404	35
25423	WH8102_3	SNP	0.0186	G	A	G	D	N	-		NA	n	404	35
25423	WH8102_4	SNP	0.0155	G	A	G	D	N	-		NA	n	404	35

25425	MED4_1	SNP	0.0578	A	G	T	A	N	-		NA	n	406	35
25425	MED4_2	SNP	0.1062	A	G	T	A	N	-		NA	n	406	35
25425	MED4_3	SNP	0.0997	A	G	T	A	N	-		NA	n	406	35
25425	MED4_4	SNP	0.9319	A	G	T	A	N	-		NA	n	406	35
25425	MED4_5	SNP	0.4043	A	G	T	A	N	-		NA	n	406	35
25425	MIT9515_1	SNP	0.1846	A	G	T	A	N	-		NA	n	406	35
25425	MIT9515_2	SNP	0.0659	A	G	T	A	N	-		NA	n	406	35
25425	MIT9515_3	SNP	0.1176	A	G	T	A	N	-		NA	n	406	35
25425	MIT9515_4	SNP	0.0696	A	G	T	A	N	-		NA	n	406	35
25425	WH8102_1	SNP	0.6446	A	G	T	A	N	-		NA	n	406	35
25425	WH8102_2	SNP	0.0336	A	G	T	A	N	-		NA	n	406	35
25425	WH8102_3	SNP	0.0542	A	G	T	A	N	-		NA	n	406	35
25425	WH8102_4	SNP	0.1283	A	G	T	A	N	-		NA	n	406	35
25577	WH8102_1	SNP	0.0154	C	G	N	K	N	-		NA	n	558	35
25714	MIT9515_1	SNP	0.3349	A	G	D	G	N	-		NA	n	695	35
25714	MIT9515_2	SNP	0.7210	A	G	D	G	N	-		NA	n	695	35
25714	MIT9515_3	SNP	0.5068	A	G	D	G	N	-		NA	n	695	35
25714	MIT9515_4	SNP	0.7725	A	G	D	G	N	-		NA	n	695	35
25714	WH8102_1	SNP	0.0210	A	G	D	G	N	-		NA	n	695	35
25714	WH8102_2	SNP	0.9699	A	G	D	G	N	-		NA	n	695	35
25714	WH8102_3	SNP	0.9066	A	G	D	G	N	-		NA	n	695	35
26092	WH8102_4	SNP	0.0523	C	T	T	I	N	-		NA	n	1073	35
34369	MED4_4	SNP	0.4286	C	G	Q	E	N	-		NA	y	802	2
34369	MED4_5	SNP	0.0938	C	G	Q	E	N	-		NA	y	802	41
59727	WH8102_2	SNP	0.0796	A	T	Q	H	N	-		NA	n	1323	100
62933	MIT9515_1	SNP	0.0267	C	T	P	L	N	-		NA	n	2495	101
62933	MIT9515_3	SNP	0.0458	C	T	P	L	N	-		NA	n	2495	101
63344	WH8102_3	SNP	0.0852	G	A	G	D	N	-		NA	n	2906	101
64775	MED4_3	SNP	0.0769	C	T	T	I	N	-		NA	n	4337	101
65494	WH8102_3	INS	0.1151	NA	-	NA	-	-	-	CTGATGGTT	9	n	5056	101
69408	WH8102_3	INS	0.0888	NA	-	NA	-	-	-	GCAGCT	6	n	8970	101
71137	WH8102_2	SNP	0.0389	C	A	Q	K	N	-		NA	n	1069 9	101
71224	MED4_1	SNP	0.0544	G	A	A	T	N	-		NA	n	1078 6	101
71245	MED4_1	SNP	0.0457	T	A	L	I	N	-		NA	n	1080	101

													7	
71437	MED4_2	SNP	0.1903	A	G	T	A	N	-		NA	n	1099<br>9	101
72154	MED4_2	SNP	0.7026	T	C	S	P	N	-		NA	n	1171<br>6	101
72178	WH8102_3	SNP	0.7998	A	G	N	D	N	-		NA	n	1174<br>0	101
84683	MED4_3	INS	0.4580	NA	-	NA	-	-	-	GGT	3	n	3174	103
84979	MED4_1	SNP	0.2451	C	T	T	I	N	-		NA	n	3470	103
84979	MED4_2	SNP	0.2276	C	T	T	I	N	-		NA	n	3470	103
84979	MED4_3	SNP	0.0582	C	T	T	I	N	-		NA	n	3470	103
84979	MED4_5	SNP	0.2825	C	T	T	I	N	-		NA	n	3470	103
84979	WH8102_3	SNP	0.0328	C	T	T	I	N	-		NA	n	3470	103
86520	MED4_3	INS	0.3490	NA	-	NA	-	-	-	ATCACT	6	n	831	104
88603	MED4_2	SNP	0.1531	A	G	D	G	N	-		NA	n	1382	105
88603	MED4_4	SNP	0.0184	A	G	D	G	N	-		NA	n	1382	105
95267	MED4_1	SNP	0.2611	C	T	P	L	N	-		NA	n	59	108
95435	WH8102_1	SNP	0.6960	G	A	G	D	N	-		NA	n	227	108
95504	MED4_1	SNP	0.0403	A	G	Q	R	N	-		NA	n	296	108
95504	MED4_2	SNP	0.0410	A	G	Q	R	N	-		NA	n	296	108
95504	MED4_3	SNP	0.0532	A	G	Q	R	N	-		NA	n	296	108
95504	WH8102_3	SNP	0.1087	A	G	Q	R	N	-		NA	n	296	108
95539	WH8102_1	SNP	0.0476	G	A	D	N	N	-		NA	n	331	108
95539	WH8102_3	SNP	0.0267	G	A	D	N	N	-		NA	n	331	108
95539	WH8102_4	SNP	0.1494	G	A	D	N	N	-		NA	n	331	108
95551	WH8102_1	SNP	0.0450	A	G	N	D	N	-		NA	n	343	108
95551	WH8102_3	SNP	0.0411	A	G	N	D	N	-		NA	n	343	108
95600	WH8102_1	SNP	0.0477	A	G	Y	C	N	-		NA	n	392	108
95600	WH8102_2	SNP	0.0244	A	G	Y	C	N	-		NA	n	392	108
95788	MED4_1	SNP	0.0204	A	G	T	A	N	-		NA	n	580	108
95788	MED4_2	SNP	0.0753	A	G	T	A	N	-		NA	n	580	108
95788	MED4_3	SNP	0.0239	A	G	T	A	N	-		NA	n	580	108
95788	MED4_4	SNP	0.0522	A	G	T	A	N	-		NA	n	580	108
95788	MED4_5	SNP	0.0211	A	G	T	A	N	-		NA	n	580	108
95788	MIT9515_1	SNP	0.1595	A	G	T	A	N	-		NA	n	580	108
95788	MIT9515_2	SNP	0.0500	A	G	T	A	N	-		NA	n	580	108

95788	MIT9515_3	SNP	0.1169	A	G	T	A	N	-		NA	n	580	108
95788	WH8102_3	SNP	0.0312	A	G	T	A	N	-		NA	n	580	108
95840	WH8102_1	SNP	0.1312	C	A	A	E	N	-		NA	n	632	108
95840	WH8102_3	SNP	0.0243	C	A	A	E	N	-		NA	n	632	108
95840	WH8102_4	SNP	0.7720	C	A	A	E	N	-		NA	n	632	108
95878	WH8102_1	SNP	0.0209	G	T	G	C	N	-		NA	n	670	108
95878	WH8102_4	SNP	0.0846	G	T	G	C	N	-		NA	n	670	108
97708	MED4_5	SNP	0.2520	T	C	Y	H	N	-		NA	n	2500	108
98959	WH8102_2	INS	0.7649	NA	-	NA	-	-	-	CAT	3	n	3751	108
99133	WH8102_2	SNP	0.2085	A	G	N	D	N	-		NA	n	3925	108
100113	WH8102_3	SNP	0.4671	T	A	T	T	Y	Y		NA	n	4905	108
100119	WH8102_3	SNP	0.4623	T	A	T	T	Y	Y		NA	n	4911	108
100122	WH8102_3	SNP	0.3988	T	C	H	H	Y	Y		NA	n	4914	108
100125	WH8102_3	SNP	0.3876	C	T	F	F	Y	N		NA	n	4917	108
100131	WH8102_3	SNP	0.3577	T	C	Y	Y	Y	Y		NA	n	4923	108
100143	WH8102_3	SNP	0.1994	C	T	I	I	Y	N		NA	n	4935	108
100149	WH8102_3	SNP	0.1915	T	C	H	H	Y	Y		NA	n	4941	108
100173	WH8102_3	SNP	0.1133	T	A	T	T	Y	Y		NA	n	4965	108
100176	WH8102_3	SNP	0.1130	G	A	Q	Q	Y	Y		NA	n	4968	108
100179	WH8102_3	SNP	0.1178	T	A	S	S	Y	Y		NA	n	4971	108
100215	WH8102_3	SNP	0.3201	T	A	T	T	Y	Y		NA	n	5007	108
100218	WH8102_3	SNP	0.3190	C	T	N	N	Y	N		NA	n	5010	108
100221	WH8102_3	SNP	0.3226	C	A	L	L	Y	?		NA	n	5013	108
100224	WH8102_3	SNP	0.3322194	T	C	F	F	Y	Y		NA	n	5016	108
100227	WH8102_3	SNP	0.3298	T	A	T	T	Y	Y		NA	n	5019	108
113504	WH8102_1	SNP	0.0126	A	T	D	V	N	-		NA	n	827	128
118159	WH8102_4	SNP	0.0745	G	T	A	S	N	-		NA	n	544	132
157342	WH8102_1	SNP	0.0437	G	A	R	H	N	-		NA	n	71	190
158613	MED4_5	SNP	0.2744	G	A	G	E	N	-		NA	n	314	192
167684	WH8102_2	DEL	0.8249	NA	-	NA	-	-	-	TCAAGGAGCATAAATAAATTATATTATTAGCATCAGGAGTTGACGATGCCAA	52	y	171	215
171638	WH8102_3	SNP	0.8707	A	T	K	*	N	-		NA	n	7	224
171666	WH8102_1	SNP	0.9581	C	A	A	D	N	-		NA	n	35	224

171666	WH8102_2	SNP	0.8972	C	A	A	D	N	-		NA	n	35	224
171666	WH8102_3	SNP	0.0736	C	A	A	D	N	-		NA	n	35	224
171666	WH8102_4	SNP	0.2267	C	A	A	D	N	-		NA	n	35	224
171712	WH8102_3	INS	0.0295	NA	-	NA	-	-	-	A	1	n	81	224
171712	WH8102_3	INS	0.0293	NA	-	NA	-	-	-	T	1	n	81	224
171803	WH8102_4	DEL	0.0136	NA	-	NA	-	-	-	ATCGGTTGGTACG TATCTGGTGGT	24	n	172	224
171817	WH8102_4	INS	0.0845	NA	-	NA	-	-	-	CAGGTGTTTCTCC	13	n	186	224
171896	WH8102_2	SNP	0.0855	T	C	S	P	N	-		NA	n	265	224
171914	WH8102_4	SNP	0.0143	G	A	G	R	N	-		NA	n	283	224
171933	WH8102_4	SNP	0.5448	C	A	T	N	N	-		NA	n	302	224
171962	WH8102_4	DEL	0.0826	NA	-	NA	-	-	-	GCTAAGGCAGGTG TTAAGTTCGTT	24	n	331	224

Supplementary Table 3: Summary of mutated genes in the S-TIM4 genome

ORF	Product	Gene name *	Coordinates	# mutated populations	# mutations	transcription phase**	MS identification
13	Base-plate wedge component [1]	gp53	4986..5945	1	1	Late	Y
23	2OG-Fe(II) oxygenase [1]	unnamed	16481..17167	2	4	Middle	N
24	Structural protein [2]	unnamed	17194..17898	13	21	No Homologue	Y
25	Hypothetical protein	unnamed	17911..18255	2	2	Late	N
27	Base-plate hub + tail lysozyme [1]	gp5	18732..21272	2	4	Middle	Y
34	Structural protein [1+2]	unnamed	23712..25013	3	3	Late	Y
35	Structural protein [1+2]	unnamed	25020..26471	13	29	Late	Y
41	Structural protein [2]	unnamed	33568..34731	2	2	No Homologue	Y
100	Base-plate wedge [2]	gp6	58405..60432	1	1	Late	Y
101	Structural protein [1+2]	unnamed	60439..80250	7	12	Late	Y
103	Structural protein [2]	unnamed	81510..85652	5	6	Late	Y
104	Base-plate wedge [1]	gp8	85690..87222	1	1	Late	Y
105	VrlC protein (similar to baseplate wedge connector proteins) [1]	unnamed	87222..94688	2	2	Late	Y
108	YadA domain-containing structural protein (related to tail fiber assembly) [1]	unnamed	95209..101919	12	45	Late	Y
128	Tail sheath monomer [1]	gp18	112678..114930	1	1	Late	Y
132	Procapsid core scaffold and protease [1]	gp21	117616..118260	1	1	Late	Y
190	Hypothetical protein [1]	unnamed	157272..157598	1	1	Late	N
192	Hypothetical protein [1]	unnamed	158300..158689	1	1	Late	N
215	Hypothetical protein	unnamed	167514..167696	1	1	No Homologue	N
224	DUF680 domain-containing protein [1]	unnamed	171632..171991	4	13	Middle	N

* Gene name is based on best BLAST homolog.
** Transcription phase predicted using homology to cyanophage Syn9 genes [2].
[1] Product prediction based on sequence homology
[2] product prediction based on MS prediction (this study)

Supplementary Table S4: Hypervariable regions, identified by recruitment of the GOS metagenome

Start (genomic position)	End (genomic position)	length
602	1334	732
1967	3039	1072
4652	5407	755
5965	6837	872
9664	12081	2417
13535	14864	1329
15829	16330	501
17460	18056	596
18464	19067	603
19979	21271	1292
33780	34465	685
35528	36345	817
37169	37708	539
39258	39781	523
40090	41571	1481
42714	44059	1345
44512	45106	594
46241	49576	3335
53739	54523	784
54726	57990	3264
93479	94017	538
107120	107799	679
129899	130760	861
136124	136653	529
151983	152515	532
153920	156114	2194
167606	168173	567

Supplementary Table 5: Summary of mutations identified in Syn19 populations.

Genome position	Population	Type	Frequency	Old base	New base	Old AA	New AA	Synonymous (Y/N)	Mutation location in the gene	ORF	Gene product
24954	1	SNP	0.80137775	G	A	G	D	N	455	33	Hypothetical-Protein
26717	1	SNP	0.01306629	G	T	D	Y	N	889	34	structural_protein
54443	1	SNP	0.13087175	C	T	S	F	N	1823	87	baseplate_wedge
56193	1	SNP	0.02844334	C	T	R	C	N	1540	88	Hypothetical-Protein
156184	1	SNP	0.1090405	C	A	L	I	N	22	183	Hypothetical-Protein
24954	2	SNP	0.50029015	G	A	G	D	N	455	33	Hypothetical-Protein
26717	2	SNP	0.09739735	G	T	D	Y	N	889	34	structural_protein
54443	2	SNP	0.28943325	C	T	S	F	N	1823	87	baseplate_wedge
56193	2	SNP	0.10569715	C	T	R	C	N	1540	88	Hypothetical-Protein
56680	2	SNP	0.01617957	C	T	P	L	N	2027	88	Hypothetical-Protein
156184	2	SNP	0.01839972	C	A	L	I	N	22	183	Hypothetical-Protein
24954	3	SNP	0.34615305	G	A	G	D	N	455	33	Hypothetical-Protein
54443	3	SNP	0.27007245	C	T	S	F	N	1823	87	baseplate_wedge
56193	3	SNP	0.3100574	C	T	R	C	N	1540	88	Hypothetical-Protein
56803	3	SNP	0.03923369	G	A	G	D	N	2150	88	Hypothetical-Protein
156184	3	SNP	0.06295562	C	A	L	I	N	22	183	Hypothetical-Protein
24954	4	SNP	0.1504424	G	A	G	D	N	455	33	Hypothetical-Protein
26717	4	SNP	0.18702885	G	T	D	Y	N	889	34	structural_protein
54443	4	SNP	0.4577372	C	T	S	F	N	1823	87	baseplate_wedge
56193	4	SNP	0.08600593	C	T	R	C	N	1540	88	Hypothetical-Protein

1 Schwartz, D. A. & Lindell, D. Genetic hurdles limit the arms race between *Prochlorococcus* and the T7-like podoviruses infecting them. *ISME J* **11**, 1836-1851, doi:10.1038/ismej.2017.47 (2017).
2 Doron, S. *et al.* Transcriptome dynamics of a broad host-range cyanophage and its hosts. *ISME J* **10**, 1437-1455, doi:10.1038/ismej.2015.210 (2016).
